# Establishing the link between motivational disturbances and behavioural rigidity in frontotemporal dementia

**DOI:** 10.1111/ene.16132

**Published:** 2023-11-07

**Authors:** Kristina Horne, Rebekah M. Ahmed, Olivier Piguet, Muireann Irish

**Affiliations:** ^1^ The University of Sydney, Brain and Mind Centre Sydney New South Wales Australia; ^2^ The University of Sydney, School of Psychology Sydney New South Wales Australia; ^3^ The University of Sydney, School of Medical Sciences Sydney New South Wales Australia; ^4^ Memory and Cognition Clinic, Department of Clinical Neurosciences Royal Prince Alfred Hospital Sydney New South Wales Australia

**Keywords:** anhedonia, apathy, frontotemporal dementia, inflexibility, semantic dementia

## Abstract

**Background:**

Rigid and inflexible behaviours are common in frontotemporal dementia (FTD), manifesting in compulsive pursuit of specific interests, routines, and rituals. Paradoxically, these changes occur alongside profound motivational disturbances including apathy and anhedonia. While posited to be related, no study to date has explored the link between motivational changes and behavioural rigidity in FTD.

**Methods:**

Carer ratings for 71 FTD participants (26 semantic dementia [SD], 45 behavioural variant [bvFTD]) were obtained on the Dimensional Apathy Scale (apathy), the Snaith‐Hamilton Pleasure Scale (hedonic tone) and the Cambridge Behavioural Inventory–Revised (CBI‐R; behavioural changes). A rigidity index was created from existing items on the CBI‐R. Whole‐brain voxel‐based morphometry was used to explore associations between rigidity and grey matter intensity in the combined FTD group.

**Results:**

Behavioural rigidity was significantly related to apathy severity (*r* = 0.57) and decreased hedonic tone (*r* = −0.36) in the combined FTD group. Multiple linear regression revealed a significant diagnosis × hedonic tone interaction (*β* = −1.40), whereby lower hedonic tone predicted rigidity in SD (*r* = −0.65) but not in bvFTD (*r* = −0.18). In contrast, the relationship between rigidity and apathy did not differ between the groups (*β* = −0.42). At the neural level, rigidity correlated with degeneration of predominantly right‐sided frontostriatal structures including, notably, the nucleus accumbens.

**Conclusions:**

As the first study to demonstrate a link between motivational changes and behavioural rigidity in FTD, our findings have important clinical implications. By identifying candidate mechanisms of behavioural rigidity, our findings can inform targeted interventions to manage inflexible patterns of thought and behaviour in daily life.

## INTRODUCTION

Decreased capacity to adjust one's thoughts and behaviours in response to changing environmental demands is a hallmark feature of frontotemporal dementia (FTD) [1]. In daily life, this rigidity manifests in the form of stereotypical, perseverative, or compulsive behaviours. While behavioural disturbances are most commonly ascribed to the behavioural variant of FTD (bvFTD), mounting evidence indicates profound inflexibility in semantic dementia (SD) [2]. In parallel with semantic knowledge impairments, SD patients display a progressive narrowing of interests [3], idiosyncratic fixations including clockwatching and timekeeping [2], as well as the emergence of intense or obsessive interests (e.g., preoccupation with colours, puzzles, and numbers) [2, 3, 4]. Increasingly rigid eating preferences and behaviours have further been documented in both bvFTD and SD syndromes [5]. Over time, this inflexibility can result in the pursuit of new, and sometimes unusual, interests, resulting in repetitive or ritualistic behaviours [6].

We recently proposed that striatally mediated changes in reward processing represents a biologically plausible mechanism that modulates inflexible behaviours in FTD [6]. Accordingly, damage to a primary hedonic pathway dampens the hedonic response to previously enjoyable activities, resulting in a progressive narrowing of activities from which individuals derive pleasure. Evidence of profound anhedonia (i.e., a diminished ability to experience pleasure) in FTD provides preliminary support for this proposal [7, 8]. In addition, loss of goal‐directed behaviour in the form of apathy is well documented in bvFTD [2] and SD [3], particularly with disease progression. These motivational disturbances likely compound habitual behaviour by impairing the ability to generate new ideas and reducing the drive to deviate from established routines.

Behavioural rigidity in FTD has received scant attention to date, and its aetiology remains unclear. The objectives of this study were therefore twofold: (i) to explore the link between inflexible behaviours and motivational changes in FTD using currently available clinical tools, and (ii) to establish the neural correlates of inflexible behaviours in FTD. In line with our theoretical model, we hypothesized that behavioural rigidity in FTD would correlate with motivational changes (i.e., anhedonia, apathy) and would reflect the degeneration of frontostriatal brain regions implicated in reward processing. We also explored whether this relationship differed between diagnostic groups, given previous findings of elevated rigidity in SD relative to bvFTD [2].

## METHODS

### Participants

A total of 71 participants were recruited through FRONTIER, the FTD research clinic at the Brain and Mind Centre, University of Sydney, Australia, including 26 participants with SD [9] and 45 participants with clinically probable bvFTD [10]. A subgroup of 54 participants (36 bvFTD, 18 SD), matched to the overall FTD sample for demographic and clinical variables, underwent 3‐T structural imaging for voxel‐based morphometry (VBM) analyses. Diagnoses were established by a senior neurologist, neuropsychologist, and occupational therapist, based on comprehensive clinical assessment and neuropsychological testing. Participants were excluded if they scored <40 on the Addenbrooke's Cognitive Examination (ACE‐III) [11] indicating severe cognitive impairment. The study was approved by the University of New South Wales Ethics Committee and the South‐Eastern Sydney Local Health District. All participants, or their carers, provided informed consent in accordance with the Declaration of Helsinki.

### Measures

#### Behavioural rigidity

In the absence of a validated instrument measuring rigidity in dementia, we derived a composite ‘rigidity index’ from selected items of the Cambridge Behavioural Inventory‐Revised (CBI‐R) [12]. Six items were identified by consensus among four FTD researchers as capturing core features of rigid thinking and behaviour (e.g., ‘Is rigid and fixed in her/his ideas and opinions’). Carer ratings on these items were entered into a principal component analysis which produced a single component solution, accounting for 52.17% of the total variance (Appendix S1).

#### Motivational disturbances

A modified version of the Snaith‐Hamilton Pleasure Scale (SHAPS) was used to index carer‐rated hedonic tone, with lower scores indicating a greater loss of hedonic tone (i.e., anhedonia) in the patient groups. The motivation subscale of the CBI was used to measure carer‐reported apathy via a percentage‐corrected score, with higher ratings indicating greater apathy in the patient groups.

### Statistical analyses

Behavioural analyses were completed in R programming software. Independent samples *t*‐tests were used to examine group differences on continuous variables (age, education, ACE‐III). Analysis of covariance (ANCOVA) was performed to explore group differences in rigidity, hedonic tone, and apathy, controlling for age and total ACE‐III score. Pearson correlations explored associations between rigidity, hedonic tone, and apathy, followed by multiple regression analyses to determine whether the identified associations differed between FTD subtypes. Whole‐brain VBM was conducted in FMRIB Software Library (FSL) using a standard processing pipeline to explore voxelwise associations between behavioural rigidity and grey matter intensity across the FTD imaging sample (*n* = 54; full details provided in Appendix S1).

## RESULTS

Participants did not differ with regard to years of education (confidence interval [CI] −1.88 to 1.31; *p* = 0.720) or sex distribution (*χ* ^2^ = 2.07, *p* = 0.150). SD patients were on average 4 years older than the bvFTD group (CI −7.87 to −0.57; *t* = 2.31, df = 69, *p* = 0.024). bvFTD patients scored significantly higher on the ACE‐III relative to SD (mean difference = 13.57, *t* = −4.50, df = 67, CI 7.55 to 19.60; *p* < 0.001 [Table 1]).

**TABLE 1:** Demographic and clinical characteristics of the study sample

	bvFTD (*n* = 45)	SD (*n* = 26)	Group difference
Age, years	62.62 (7.98)	66.85 (6.35)	Significant^a^
Education, years	12.75 (3.34)	12.46 (3.08)	n/s
Sex, male: female	11:15	27:18	n/s
ACE‐III [100]	76.09 (12.26)	62.52 (11.67)	Significant^a^
CBI‐R Rigidity Index [24]	10.32 (5.87)	9.81 (6.32)	n/s
CBI‐R Motivation [100]	57.92 (28.88)	38.46 (26.86)	Significant^a^
SHAPS [56]	39.93 (7.43)	40.96 (7.44)	n/s

*Note*: Values presented as mean (standard deviation) unless otherwise specified. Maximum score for each test shown in square brackets where appropriate.

Abbreviations: ACE‐III, Addenbrooke's Cognitive Examination III; bvFTD, behavioural variant frontotemporal dementia; CBI‐R, Cambridge Behavioural Inventory—Revised; n/s, not significant; SD, semantic dementia; SHAPS, Snaith‐Hamilton Pleasure Scale.

^a^ Significant group difference at *p* < 0.05.

An ANCOVA controlling for age and ACE‐III revealed comparable levels of rigidity (CBI Rigidity Index: CI −5.71 to 1.46; *p* = 0.651) and hedonic tone (SHAPS: CI −1.13 to 7.41; *p* = 0.556) in bvFTD and SD. Apathy, however, was significantly higher in bvFTD relative to SD (CBI‐R Motivation: mean difference = 28.35, *t* = −3.52, df = 69, CI −44.42 to −12.28; *p* < 0.001).

Pearson R correlations across the FTD sample revealed that rigidity significantly correlated with motivational disturbances, whereby higher levels of rigidity were associated with lower levels of hedonic tone (*r* = −0.36, CI −0.55 to −0.14; *p* = 0.002) and higher levels of apathy (*r* = 0.57, CI 0.39 to 0.71; *p* < 0.001).

**FIGURE 1:**
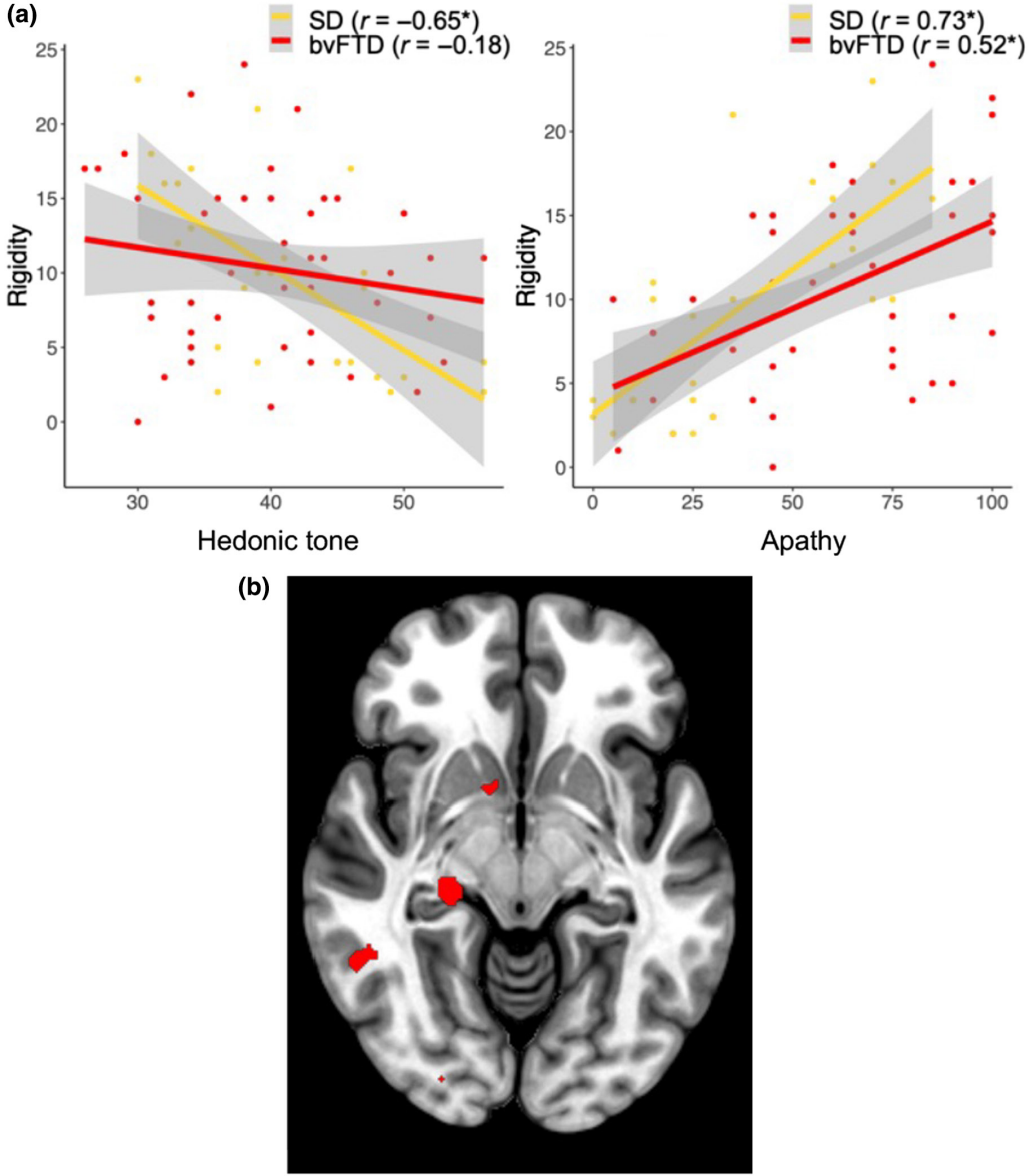
Behavioural correlations (top panels) and voxel‐based morphometry results (bottom panel) demonstrating associations between behavioural rigidity and motivational disturbances in frontotemporal dementia (FTD). (a) Associations between behavioural rigidity and reduced hedonic tone (left) and apathy (right) for semantic dementia (SD; yellow) and behavioural‐variant FTD (bvFTD; red). Shaded areas depict 95% confidence intervals. *Denotes significant correlation at *p* <0.05. (b) Voxel‐based morphometry results in the combined FTD sample displaying significant associations between behavioural rigidity and grey matter intensity decrease in the right nucleus accumbens, right hippocampus, and right temporal cortex. Results extracted voxelwise, corrected for false discovery rate at *q* = 0.05. Coordinates refer to Montreal Neurological Institute (MNI) space.

A multiple linear regression revealed a significant diagnosis × hedonic tone interaction indicating that the relationship between hedonic tone and rigidity differed between diagnostic groups (β = −1.40, CI −0.79 to 0.04; *p* = 0.030). While lower hedonic tone significantly predicted rigidity in SD (*r* = −0.65, CI −0.35 to −0.83; *p* < 0.001) this was not observed in bvFTD (*r* = −0.177, CI −0.45 to 0.13; *p* = 0.250 [Figure 1a]).

When apathy was entered as a predictor instead of hedonic tone, we did not find a significant diagnosis × apathy interaction (*β* = 0.28, CI −0.02 to 0.16; *p* = 0.13), indicating that the relationship between apathy and rigidity was comparable across FTD groups (Figure 1a).

### Voxel‐based morphometry analyses

Significant associations were found between behavioural rigidity and grey matter intensity decrease in a predominantly right lateralized brain network (Figure 1b). Key regions implicated in behavioural rigidity included the superior frontal gyrus and frontal pole, right hippocampus extending to the right parahippocampal gyrus, right posterior middle temporal gyrus, and right nucleus accumbens.

## DISCUSSION

The objective of this study was to explore a putative association between inflexible behaviours in FTD and concomitant changes in motivation. For the first time, we demonstrate that behavioural rigidity is a common feature in both bvFTD and SD and one that appears closely linked to changes in motivation. Importantly, linear regression analyses within each patient group hinted at disease‐specific differences in the origin of behavioural rigidity. While apathy emerged as a predictor of inflexible behaviours in both bvFTD and SD, a reduction in hedonic tone was exclusively associated with behavioural rigidity in SD. Our findings have important clinical implications for the identification and management of behavioural disturbances in people living with FTD.

Patients with FTD have been observed to display somewhat paradoxical patterns of behaviour, whereby a generalized apathetic profile can coexist with intense reward‐seeking behaviours directed at unusual and sometimes non‐productive targets [13]. That apathy and a reduction in hedonic tone related to rigid behaviours in FTD makes intuitive sense in light of current theoretical frameworks of motivation. For example, effort avoidance has been proposed as a core mechanism of apathy in bvFTD [14]. Accordingly, habitual behaviours in bvFTD may represent a path of least resistance, enabling the individual to circumvent the mental effort required to generate alternate ideas or to switch their behaviours from established routines [1]. In contrast, compulsive and ritualistic behaviours in SD have been posited to reflect a narrowing of interests to a few residual targets from which pleasure can be derived [6]. Our findings provide the first empirical support for this position and suggest that alterations in core hedonic processing amplify the proclivity for routinized and stereotypical behaviours in SD. This truncating of interests likely biases the individual towards a handful of specific activities that remain familiar, which are then pursued with vigour [6, 13].

Whole‐brain structural neuroimaging revealed significant associations between inflexible behaviour and changes in regions typically implicated in reward processing, memory, and semantics. Across the FTD group, increased behavioural rigidity was associated with a predominantly right‐lateralized network of cortical and subcortical regions, including the right nucleus accumbens, right hippocampus, and right temporal neocortex. Many of these structures have previously been implicated in eating behaviour changes in FTD [5]. Our study provides converging behavioural and neuroimaging evidence to suggest that inflexible behaviours in FTD are closely tied to alterations in established reward pathways.

As the first study to empirically demonstrate a link between inflexible behaviours and motivational disturbances in FTD, we note many opportunities for future research. A priority should be the development of validated assessments to comprehensively characterize the multidimensional nature of rigidity and its varied expression depending on contextual, social, and individual factors. Similarly, it will be essential to understand how apathy and anhedonia influence inflexible behaviours at different stages of the FTD disease course and to investigate how these profiles potentially differ across clinical phenotypes. Recruitment of larger samples will further enable us to elucidate disease‐specific mechanisms of rigidity at the neural level. Longitudinal approaches will prove particularly important in this regard. Future studies incorporating ecologically valid behavioural measures of motivation and rigidity could mitigate against potential biases inherent in carer report.

We anticipate that these findings will stimulate future research to determine potential drivers of behavioural rigidity in FTD. Ultimately, we envisage that this work will inform the development of effective strategies to manage this overlooked clinical symptom, leading to enhanced outcomes for patients and their carers.

## AUTHOR CONTRIBUTIONS

**Kristina Horne:** Conceptualization; writing – original draft; writing – review and editing; formal analysis. **Rebekah M. Ahmed:** Resources; writing – review and editing. **Olivier Piguet:** Funding acquisition; writing – review and editing; project administration; resources. **Muireann Irish:** Supervision; resources; formal analysis; writing – review and editing; writing – original draft; funding acquisition; conceptualization.

## FUNDING INFORMATION

This work was supported in part by funding to ForeFront, a collaborative research group dedicated to the study of FTD and motor neuron disease, from the National Health and Medical Research Council (NHMRC; GNT1037746) and the Australian Research Council (ARC) Centre of Excellence in Cognition and its Disorders Memory Program (CE11000102). Olivier Piguet is supported in part by an NHMRC Investigator Leadership Fellowship (GNT2008020). Muireann Irish is supported by a Medical Research Future Fund Dementia, Aging, and Aged Care Grant from the Australian Government Department of Health and Aged Care (GNT2024329) and by an ARC Discovery Project (DP220100663).

## CONFLICT OF INTEREST STATEMENT

The authors have no competing interests to declare.

## Supporting information


Appendix S1

## Data Availability

The ethical requirement to ensure patient confidentiality precludes public archiving of our data. Researchers who would like to access the raw data should contact the corresponding author who will liaise with the ethics committee that approved the study, and accordingly, as much data that are required to reproduce the results will be released to the individual researcher.
