# Supplementary material for: Establishing the link between motivational disturbances and behavioural rigidity in frontotemporal dementia
Source: Eur J Neurol. 2023 Nov 7;31(2):e16132. doi: 10.1111/ene.16132 (PMC11235754; doi:10.1111/ene.16132)
Supplement: Supplementary file 1 — Appendix S1 [file ENE-31-e16132-s001.docx]

**Establishing the link between motivational disturbances and behavioural rigidity in frontotemporal dementia**

**Supplementary Material**

Kristina Horne^1,2^, Rebekah M. Ahmed^1,3,4^, Olivier Piguet^1,2^, Muireann Irish^1,2^

1. The University of Sydney, Brain and Mind Centre, Sydney, New South Wales, Australia
2. The University of Sydney, School of Psychology, Sydney, New South Wales, Australia
3. The University of Sydney, School of Medical Sciences, Sydney, New South Wales, Australia
4. Memory and Cognition Clinic, Department of Clinical Neurosciences, Royal Prince Alfred Hospital, Sydney, Australia

Files included:

1. **Supplementary Methods: Principal Components Analysis to create the Rigidity Index**
2. **Supplementary Methods: Testing for normality**
3. **Supplementary Methods: Structural neuroimaging pipeline**
4. **Supplementary Results: Covariate analyses**
5. **References**

**Supplementary Methods**

**1. Principal Components Analysis to create a Rigidity Index**

Following multidisciplinary consensus, six items from the Cambridge Behavioural Inventory–Revised (CBI-R) were identified as potentially tapping into core dimensions of behavioural rigidity typically observed in FTD:

- CBI20: Is uncooperative when asked to do something.
- CBI32: Wants to eat the same foods repeatedly.
- CBI37: Is rigid and fixed in her/his ideas and opinions.
- CBI38: Develops routines from which s/he can not easily be discouraged e.g., wanting to eat or go for walks at fixed times.
- CBI39: Clock watches or appears pre-occupied with time.
- CBI40: Repeatedly uses the same expression or catch phrase.

Carer ratings across each of these six items were extracted for the participant cohort and entered into a Principal Components Analysis (PCA) to determine whether they mapped onto a single underlying “rigidity” construct. PCA was run in RStudio (R Core Team, 2022), using FactoMineR (Lê et al., 2008) and factoextra (Kassambara & Mundt, 2017) packages. In keeping with standard PCA interpretation, factors were retained if they achieved an Eigenvalue greater than 1, indicating that the principal component accounts for more variance than the original variables (Jolliffe, 2002).

Our PCA generated a single component solution with an Eigenvalue of 3.07, which accounted for 51.11% percent of the variance. This suggested that these items are best understood as reflecting a single latent factor. All items loaded heavily (i.e., loadings > 0.5) on this rigidity component.

**2. Testing for normality**

Shapiro-Wilk’s tests were run to explore normality of distributions. All variables were normally distributed (all *p* values > 0.05), except the CBI-R Motivation score for the bvFTD group (*p* = 0.04). However, assumptions of multivariate normality were met for correlations (rigidity x hedonic tone: *p* = 0.59; rigidity x apathy: *p* = 0.76), and normality of residuals was upheld in linear regressions (Rigidity and hedonic tone regression: *p* = 087; Rigidity and apathy regression: *p* = 0.66).

**3. Structural neuroimaging pipeline**

**Image acquisition**

A subgroup of 54 participants underwent whole-brain T1-weighted structural MRI on a 3T MRI scanner equipped with a standard quadrature 8-channel head coil. Images were acquired with a 256x256 mm imaging matrix, 1mm isotropic voxel resolution, repetition time of 5.8ms and flip angle of 8°. Scans were visually inspected for image quality, head movement and white matter hyperintensities. All scans were deemed suitable for inclusion.

Prior to January 2017, scans were acquired on a Phillips scanner at Neuroscience Research Australia (NeuRA; n = 19), while those obtained from 2017 onwards were acquired on a GE Discovery MR750 scanner at the Brain and Mind Centre (n = 35). This change in scanner type occurred due to the relocation of the FRONTIER research group to the University of Sydney in January, 2017.

**Voxel-based morphometry**

Whole brain voxel-based morphometry (VBM) was used to investigate potential associations between behavioural rigidity (CBI-R rigidity index) and grey matter intensity on a voxel-wise basis. Analyses were completed in FSL (FMRIB Software Library: https://fsl.fmrib.ox.ac.uk/fsl/fslwiki) using a standard preprocessing pipeline, including brain extraction (Smith, 2002), tissue segmentation (Zhang et al., 2001) and alignment of brain-extracted images to Montreal Neurological Institute (MNI) standard space using a non-linear approach (Andersson et al., 2007a, 2007b). For all covariate analyses a negative contrast was used enabling us to explore how higher behavioural rigidity scores relate to decreased grey matter intensity in the FTD sample. Significant clusters were extracted voxelwise corrected for False Discovery Rate (FDR; *q* < 0.05). A cluster extent threshold of 10 contiguous voxels was employed to enable us to detect significant brain-behaviour associations in smaller subcortical regions. Significant results were overlaid on a standard MNI brain to obtain maximum coordinates in MNI stereotaxic space.

**4. Supplementary Methods: Covariate analyses**

To complement the main analyses, we ran a series of further ANCOVAs to control for age, ACE-III and sex. Consistent with our primary analyses, there was no significant between-group difference in rigidity (CBI Rigidity Index: CI -1.52 to 5.66, *p*=0.651), or hedonic tone (SHAPS: CI -7.36 to 1.17, *p*=0.555). As before, apathy was significantly higher in bvFTD relative to SD (CBI-R Motivation: Mean difference=-28.177, *t*=3.52, df=69, CI -44.16 to -12.19, *p*<0.001).

Partial Pearson R correlations, controlling for age, ACE-III, and sex, revealed significant associations between higher levels of behavioural rigidity and reduction in hedonic tone (*r*=-0.32, CI -0.53 to -0.073, *p* = 0.011). Similarly, higher levels of behavioural rigidity were associated with greater apathy severity (*r* = 0.56, CI 0.37 to 0.72, *p* <0.001). These results are consistent with our original analyses.

Finally, we repeated our multiple linear regressions with age, ACE-III, and sex included as covariates to ensure that our findings could not be attributed to demographic variables. As before, this revealed a significant diagnosis x hedonic tone interaction indicating that the relationship between decreased hedonic tone and behavioural rigidity differed between the diagnostic groups (β=-1.46, CI -0.82 to -0.04, *p*=0.032). When apathy was entered as a predictor instead of hedonic tone, we again found no significant diagnosis x apathy interaction (β=0.27, CI -0.03 to 0.16, *p*=0.156). These results mirror our original analyses.

**References**

Andersson, J. L., Jenkinson, M., & Smith, S. (2007a). Non-linear optimisation. FMRIB technical report TR07JA1. *Practice*.

Andersson, J. L., Jenkinson, M., & Smith, S. (2007b). Non-linear registration, aka Spatial normalisation FMRIB technical report TR07JA2. *FMRIB Analysis Group of the University of Oxford*, *2*(1), e21.

Jolliffe, I. T. (2002). *Principal component analysis for special types of data*. Springer.

Kassambara, A., & Mundt, F. (2017). Package ‘factoextra’. *Extract and visualize the results of multivariate data analyses*, *76*(2).

Lê, S., Josse, J., & Husson, F. (2008). FactoMineR: an R package for multivariate analysis. *Journal of statistical software*, *25*, 1-18.

Smith, S. M. (2002). Fast robust automated brain extraction. *Human Brain Mapping*, *17*(3), 143-155.

Zhang, Y., Brady, M., & Smith, S. (2001). Segmentation of brain MR images through a hidden Markov random field model and the expectation-maximization algorithm. *IEEE transactions on medical imaging*, *20*(1), 45-57.
